# TIGIT: A promising target to overcome the barrier of immunotherapy in hematological malignancies

**DOI:** 10.3389/fonc.2022.1091782

**Published:** 2022-12-20

**Authors:** Shenhe Jin, Ye Zhang, Fengping Zhou, Xiaochang Chen, Jianpeng Sheng, Jin Zhang

**Affiliations:** ^1^ Department of Hematology, Sir Run Run Shaw Hospital, College of Medicine, Zhejiang University, Hangzhou, China; ^2^ School of Biological Sciences, Nanyang Technological University, Singapore, Singapore

**Keywords:** TIGIT, immunotherapy, hematological malignancy, lymphoma, multiple myeloma, leukemia

## Abstract

Immune evasion through up-regulating checkpoint inhibitory receptors on T cells plays an essential role in tumor initiation and progression. Therefore, immunotherapy, including immune checkpoint inhibitor targeting programmed cell death protein 1 (PD-1) and chimeric antigen receptor T cell (CAR-T) therapy, has become a promising strategy for hematological malignancies. T cell immunoreceptor with immunoglobulin and ITIM domain (TIGIT) is a novel checkpoint inhibitory receptor expressed on immune cells, including cytotoxic T cells, regulatory T cells, and NK cells. TIGIT participates in immune regulation *via* binding to its ligand CD155. Blockage of TIGIT has provided evidence of considerable efficacy in solid tumors in preclinical research and clinical trials, especially when combined with PD-1 inhibition. However, the mechanism and function of TIGIT in hematological malignancies have not been comprehensively studied. In this review, we focus on the role of TIGIT in hematological malignancies and discuss therapeutic strategies targeting TIGIT, which may provide a promising immunotherapy target for hematological malignancies.

## Introduction

Hematological malignancy is a group of clonal malignant diseases of the hemopoietic system with highly invasive potential and heterogeneity. Despite those treatments, including chemotherapy and stem cell transplantation, improving survival, some patients still experience disease relapse without long-term survival, partly due to tumor evasion from immune recognition and killing by effector cells (1–3). In recent years, immune checkpoint blockade (ICB) therapy targeting cytotoxic T lymphocyte-associated protein 4 (CTLA-4) or programmed cell death protein 1 (PD-1), and chimeric antigen receptor T cell (CAR-T) therapy utilizing genetic engineering to alter T cells to produce transmembrane proteins on the cell surface with an extracellular antibody fragment domain that recognizes tumor antigen, brings a new direction for cancer immunotherapy (4–6). Although anti-PD-1 monoclonal antibodies (mAbs) and CAR-T therapies have been actively applied in relapsed and refractory lymphoma, multiple myeloma (MM), and leukemia, which also achieved remarkable success in some cases, a part of patients still have no response to these therapies (7–10). Therefore, in-depth research on immune checkpoint molecules’ interaction mechanisms and the discovery of novel target to overcome the barrier of immunotherapy are necessary. In addition, immune checkpoint inhibitor-related toxicity is another challenge. For example, Quagliariello reported that nivolumab and pembrolizumab would induce cardiotoxicity by increasing the inflammation of cardiomyocytes (11, 12). T cell immunoglobulin and ITIM domain (TIGIT), another inhibitory immune checkpoint molecule, has emerged as a potential target in cancer immunotherapy (13, 14). In this review, we focus on the immunomodulatory role and mechanism of TIGIT, discuss its potential as an immune target in hematological malignancies.

## TIGIT structure and its ligands

TIGIT, also named as V-set and immunoglobulin domain-containing protein 9 (VSIG9), V-set and transmembrane do-maincontaining protein 3 (VSTM3) and Washington University cell adhesion molecule (WUCAM), is a co-inhibitory molecule belonging to the immunoglobulin superfamily that was first discovered in 2009 (15–17). It consists of an extracellular immunoglobulin variable (IgV) domain, a type I transmembrane domain and an intracellular domain with an immunoreceptor tyrosine-based inhibitory motif (ITIM) and an immunoglobulin tail tyrosine (ITT)-like motif (18). TIGIT is exclusively expressed on natural killer (NK) cells and T cells, including CD8+ T cells, CD4+ T cells, and regulatory T cells (Tregs) (19, 20).

**Figure 1:**
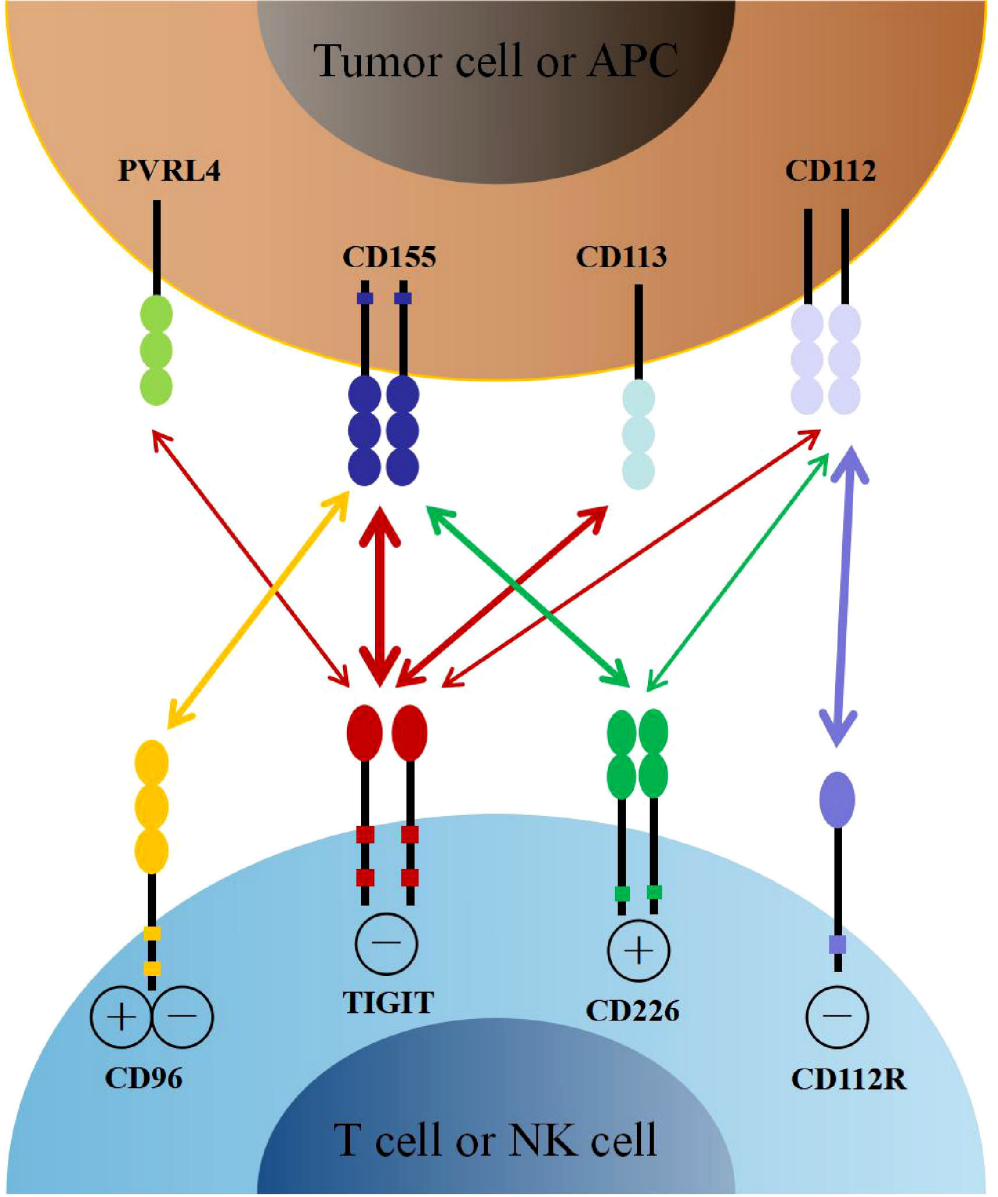
The interaction of TIGIT family receptors and ligands. TIGIT, CD226, CD96 and CD112R are expressed on T cells and NK cells. The ligands CD155, CD113, CD112 and PVRL4 are expressed on tumor cells or APCs. TIGIT delivers inhibitory signals by binding to CD155, CD113, CD112 and PVRL4, with highest affinity for CD155. CD226 and CD96 compete with TIGIT for binding to CD155, but with lower affinity than TIGIT. CD226 delivers activating signals. However, whether CD96 triggers inhibitory or activating signals remains to be determined. CD112R and CD226 also competitively binding to CD112, with higher affinity with CD112R. APCs, antigen presenting cells.

The relationship between TIGIT, its ligands, and competitive receptors is complex. On the one hand, the immunoglobulin variable domain of TIGIT shares sequence homology with members of the polio virus receptors (PVR) family, including CD155 (also named as Necl-5 or PVR), CD112 (also named as Nectin-2 or PVRL2), CD113 (also named as Nectin-3 or PVRL3), and Nectin-4 (PVRL4) (21, 22). CD155 is a member of the immunoglobulin superfamily, mainly expressed on dendritic cells (DCs), macrophages, and lymphocytes. CD112 belongs to single-pass type-I membrane glycoproteins, which is expressed on DCs and monocytes. Interestingly, CD155 and CD112 are over-expressed on different cancer cells as reported recently (23–26). In addition, CD155 has a higher affinity than CD112 to TIGIT, which became the primary ligand for TIGIT (27). By interacting with its ligands, TIGIT participates in the regulation of cellular immune function. On the other hand, TIGIT shares these ligands with other receptors, including CD226 (DNAM-1) and CD96 (TACTILE). As the costimulatory receptor, CD226 competes with TIGIT for binding to CD155 in spite of its lower affinity (27). Furthermore, CD226 also competes with TIGIT and CD112R (PVRIG) for binding to CD112 (28, 29). Therefore, CD226 also plays an essential role in immune regulation (**Figure 1**).

## Function and mechanism of TIGIT in immune regulation

Through complex interaction with ligands, TIGIT family receptors transfer inhibitory signals to immune cells, contributing to innate and adaptive immunity regulation (30–32). On the one hand, TIGIT inhibits the activity of T cells intrinsically. Firstly, TIGIT binds to CD155 and transmits intracellular inhibitory signals, directly suppressing T cell receptor (TCR) expression and signaling. Engagement of TIGIT induces down-regulation of the TCRα chain and molecules that comprise the TCR complex, as well as reduction of TCR-induced p-ERK signaling and interferon-γ (IFNγ) production in CD8+ T cells (33, 34). Secondly, TIGIT possesses a higher affinity of CD155 when competing with its costimulatory counterpart CD226, which impaires T cell function by either directly disrupting homodimerization of CD226 or decreasing expression of T-bet and production of IFNγ (35, 36).

**Figure 2:**
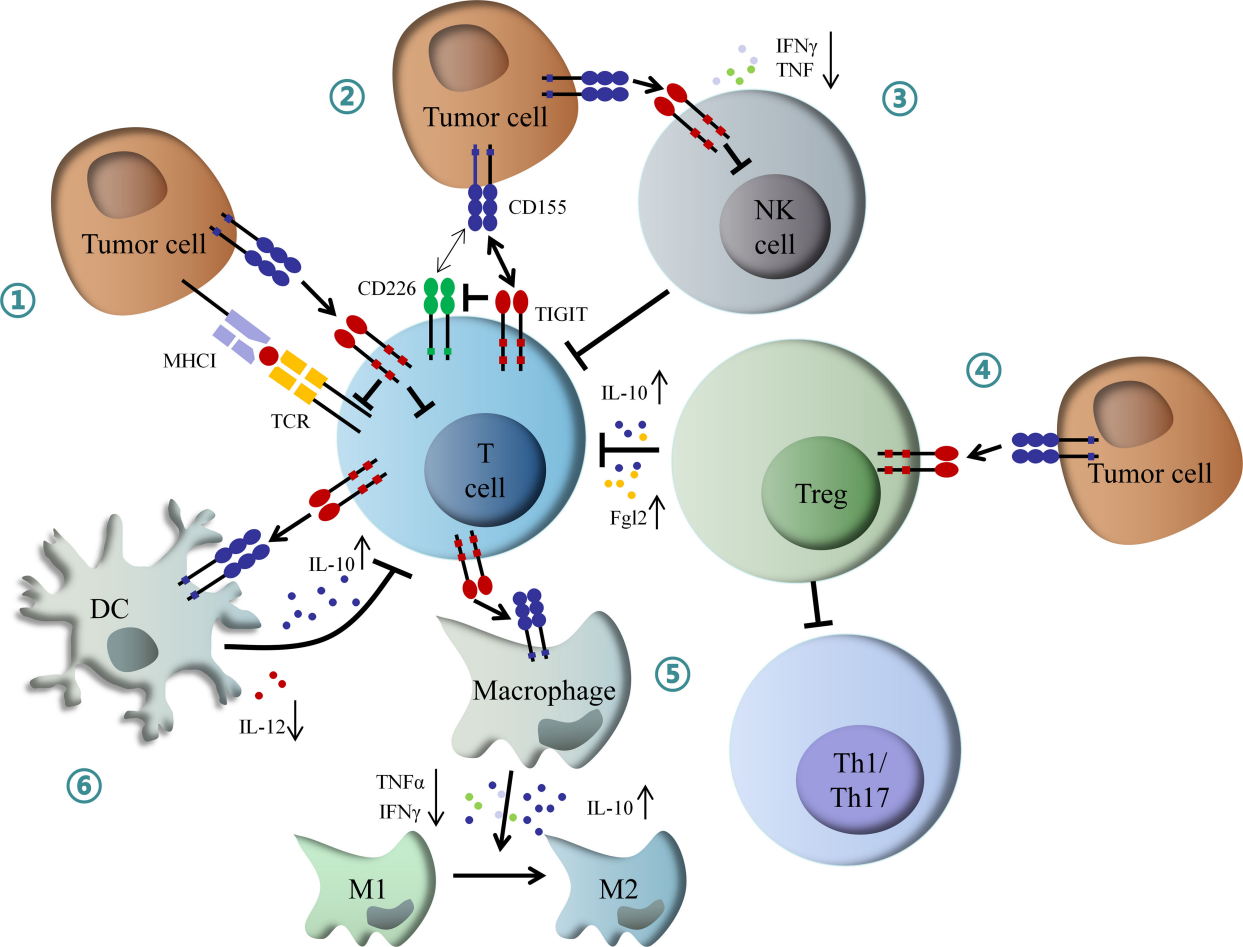
Mechanism of inhibitory role of TIGIT in immunoregulation. (1) Interaction of TIGIT with CD155 transmits intracellular inhibitory signals, which directly suppressed TCR signal and effector T cell function. (2) TIGIT inhibits CD226-induced T cell activation by disrupting CD226 homodimerization and decreasing IFNγ production. (3) TIGIT directly induces NK cell exhaustion, contributing to inactivation of CD8+ T cell. (4) TIGIT enhances Tregs mediated dysfunction of effector T cell by increased IL-10 and Fgl2, as well as inhibition of Th1 and Th17 cells. (5) TIGIT promotes macrophage switching from pro-inflammatory M1 to anti-inflammatory M2 phenotype through increased IL-10 and decreased IFNγ and TNFα. (6) TIGIT inhibits T cell function by DCs-mediated abnormal secretion of cytokines. IFNγ, interferon-γ; IL-10, interleukin-10; Fgl2, fibrinogen-like protein 2; Th1, T helper 1 cell; Th17, T helper 17 cell; TNFα, tumor necrosis factor α; DCs, dendritic cells; NK, natural killer.

On the other hand, TIGIT can exogenously enhance the immunosuppressive functions of Treg cells. TIGIT is enriched in Treg cells, which is associated with the suppressive capacity of effector T cells. Conversely, CD226 inhibits the expansion of Treg cells and promotes the secretion of IFNγ and other effector cytokines (37, 38). TIGIT expression on Treg cells also suppresses the proliferation of effector T cells *via* increased production of interleukin-10 (IL-10) and fibrinogen-like protein 2 (Fgl2), as well as the response of pro-inflammatory T helper 1 (Th1) and Th17 cells, but not Th2 cells (39). Besides, TIGIT can suppress T cell activation through DCs and macrophage-mediated cytokines disturbance. TIGIT interacts with CD155 expressed on DCs, and induces phosphorylation of CD155 through extracellular signal-regulated kinase (ERK) signaling, consequently increasing the production of anti-inflammatory cytokine IL-10 and decreasing pro-inflammatory cytokine IL-12, which inhibits T cell function (40). TIGIT also enhances the secretion of IL-10 and reduces the secretion of IFNγ and tumor necrosis factor α (TNFα) *via* c-Maf nuclear translocation, which switches macrophages from M1 to anti-inflammatory M2 phenotype (41). In addition, TIGIT also directly induces exhaustion of tumor-infiltrating NK cells with lower expression of IFNγ and TNF or indirectly contributes to exhaustion of CD8+ T cells, impairing anti-tumor immune response (42) (**Figure 2**).

## TIGIT expression and prognostic role in hematological malignancies

Increasing evidence has demonstrated that TIGIT was highly expressed on tumor-infiltrating lymphocytes (TILs) in different hematological malignancies, resulting in tumor progression and poor outcomes. Josefsson reported that TIGHT expression was significantly higher in T cells of follicular lymphoma (FL) than in healthy controls. Besides, up-regulation of TIGIT was associated with the advanced disease stage (34, 43). Yang also reported that TIGIT expression was increased on TILs in FL, and TIGIT+ T cells predicted worse treatment response and inferior survival (44). Likewise, CD4+ TIGIT+ T cells were increased in chronic lymphocytic leukemia (CLL) patients, which was also correlated with unmutated immunoglobulin heavy chain variable region (IgHv) and advanced stage (45).

Furthermore, TIGIT was similarly up-regulated in classic Hodgkin lymphoma (cHL) and Sezary syndrome (46, 47). In acute myeloid leukemia (AML) patients, elevated TIGIT expression on CD8+ T cells was observed. High TIGIT expression was associated with primary refractory disease and relapse after allogeneic stem cell transplantation (allo-SCT) with poorer survival (48, 49). TIGIT was also reported to be increased on γδ T cells and NK cells, which became an independent risk factor for prognosis (50–52). In addition, increasing frequency of TIGIT on CD8+ T cells was reported in mice models of newly diagnosed and relapsed multiple myeloma, which positively correlated with tumor burden (53, 54). These studies indicated a negative role of TIGIT in anti-tumor immunity. Therefore, targeting TIGIT may be an effective approach for ICB therapy in hematological malignancies.

## Immunotherapy targeting TIGIT in hematological malignancies

To date, immunotherapy targeting TIGIT has shown significant anti-tumor effects in several pieces of research. Catakovic reported that blockage of TIGIT by recombinant TIGIT-Fc would reduce CLL viability *in vitro* due to decreasing production of pro-survival cytokines IL-10 (45). In AML, TIGIT expression inhibited cytokine production and induced apoptosis of CD8+ T cells. Knockdown of TIGIT by siRNA could restore T cell function *via* decreasing susceptibility to apoptosis, simultaneously increasing production of TNFα and IFNγ. Besides, blockage of TIGIT significantly increased IFNγ production and NK cell degranulation, contributing to NK cells mediated anti-leukemia effects (52, 55).

Similarly, high TIGIT expression promoted T cells exhaustion, leading to myeloma progression. Conversely, the anti-TIGIT treatment prevented T cells exhaustion, decreased growth rate of tumor cells, and prolonged survival of MM mice (53). Guillerey also reported that either TIGIT deficiency or blockage by mAbs restored the immune function of anti-MM CD8+ T cells and improved survival *in vivo* (54).

In addition, dual blockade of TIGIT and PD-1 showed potential synergistic immune killing effects. On the one hand, Wang observed a higher frequency of TIGIT and PD-1 dual expression in AML patients, which was associated with a higher frequency of FLT3-ITD mutation and a lower remission rate (56). Studies showed that 68-84% of T cells had co-expression of TIGIT and PD-1 in hodgkin lymphoma (HL) (46, 57). A high frequency of TIGIT and PD-1 dual expression was also observed in CLL and FL (34, 58). On the other hand, Zhang reported that blockage of TIGIT alone only up-regulated TNFα in TIGIT+ CD4+ T cells and IFNγ, TNFα in TIGIT+ CD8+ T cells. However, combined inhibition of TIGIT, PD-1, and Tim-3 significantly up-regulated IL-2, IFNγ, and TNFα in both CD4+ and CD8+ T cells, which may enhance anti-leukemia immune responses (59). Based on the remarkable efficacy of anti-TIGIT mAbs in solid tumors and potential immune-killing effects mentioned above in preclinical research, human anti-TIGIT mAbs are being tested in phase 1/2 clinical trials either as a monotherapy or, in most studies, in combination with anti-PD-1/PD-L1 antibodies or chemotherapies for the treatment of malignant lymphoma and multiple myeloma (**Table 1**). In summary, these researches supported the progress of immunotherapy targeting the TIGIT axis in hematological malignancies.

**Table 1:** Ongoing clinical trials targeting TIGIT in hematological malignancies.

NCT number	Agent	Treatment	Tumor type	Phase
NCT05315713	Tiragolumab	Combined with mosunetuzumab ± atezolizumab (anti-PD-L1 mAb)	r/r-DLBCL r/r-FL	Phase 1/2
NCT04045028	Tiragolumab	Monotherapy Combined with rituximab Combined with daratumumab ± atezolizumab	r/r-B-NHL r/r-MM	Phase 1
NCT05267054	Ociperlimab (BGB A1217)	Combined with rituximab Combined with tislelizumab (anti-PD-1 mAb)	r/r-DLBCL	Phase 1/2
NCT04150965	BMS-986207	Combined with pomalidomide and dexamethasone	r/r-MM	Phase 1/2
NCT05005442	Vibostolimab (MK7684A)	Combined with pembrolizumab (anti-PD-1 mAb)	r/r-HL r/r-B-NHL r/r-MM	Phase 2
NCT04354246	COM902	Monotherapy Combined with COM701 (anti-PVRIG mAb)	MM	Phase 1
NCT04254107	SEA-TGT	Monotherapy Combined with sasanlimab (anti-PD-1 mAb)	cHL DLBCL PTCL-NOS	Phase 1
NCT04772989	AB308	Combined with zimberelimab (anti-PD-1 mAb)	DLBCL MM	Phase 1
NCT05289492	EOS-448	Monotherapy Combined with iberdomide ± dexamethasone	r/r-MM	Phase 1/2

r/r-DLBCL, relapsed or refractory diffuse large B cell lymphoma; r/r-FL, relapsed or refractory follicular lymphoma; r/r-B-NHL, relapsed or refractory B cell non-hodgkin’s lymphoma; r/r-MM, relapsed or refractory multiple myeloma; r/r-HL, relapsed or refractory hodgkin’s lymphoma; PTC-NOS, peripheral T-cell lymphoma, not otherwise specified; cHL, classical hodgkin’s lymphoma.

## Toxicities of TIGIT blockage

Even though therapeutic strategy targeting TIGIT has provided evidence of encouraging efficacy in hematological malignancies, the immune-related adverse events (irAEs) mediated by over-activated T cells may result in multiple organ dysfunction and poor prognosis. Phase 1 study of the anti-TIGIT antibody vibostolimab reported that two patients suffered irAEs, including one adrenal insufficiency and one severe skin reaction (60). Another phase 1 study of anti-TIGIT antibody ociperlimab in combination with anti-PD-1 antibody tislelizumab in advanced solid tumors showed that 15 of 26 patients suffered irAEs, including three severe irAEs (grade≥3) (61). CITYSCAPE trial also reported that 69% of patients experienced irAEs after treated with anti-TIGIT antibody tiragolumab and anti-PD-L1 antibody atezolizumab, in which skin rash was the most common, followed by pancreatitis, hypothyroidism, colitis and diabetes mellitus (62). Therefore, clinicians should pay more attention to the immune toxicity of anti-TIGIT therapy.

## Conclusion

The immune checkpoint molecule TIGIT plays an inhibitory role in anti-tumor immunity by inactivating immune effector cells. Up-regulation of TIGIT has been reported in various hematological malignancies, which predicts poor outcomes. Preclinical research has demonstrated that blocking TIGIT alone or combined with PD-1 improves anti-tumor immune responses. The clinical evidence of its efficacy in ongoing clinical trials, especially synergized with other immune checkpoint inhibitors, for example PD-1, lymphocyte activation gene 3 (LAG-3) and T-cell immunoglobulin-3 (TIM-3), is eagerly awaited. Furthermore, simultanously blockade of TIGIT and hypoxia-inducible factor 1-alpha (HIF-1α) may also become a potential treatment strategy (63). In the future, a comprehensive understanding of the intricate immunoregulatory network among TIGIT family members and other immune checkpoint molecules may provide more effective options for patients with hematological malignancies.

## Author contributions

JZ contributed to the conception of this review, SJ and YZ were responsible for screening literatures and drafting the manuscript, FZ, XC, and JS edited tables and figures. All authors revised the manuscript and JZ gave the final approval of the manuscript.
